# Active search for tuberculosis in three youth detention centers in Peru

**DOI:** 10.17843/rpmesp.2024.414.13727

**Published:** 2024-11-29

**Authors:** Judith Jimenez, Ana Karina Millones, Daniela Puma, Jesús Peinado, Blanca Martínez, Marco Tovar, Leonid Lecca

**Affiliations:** 1 Partners In Health, Peru Office. Lima, Peru. Partners In Health, Peru Office Lima Peru; 2 Faculty of Health Sciences, School of Medicine, Universidad Peruana de Ciencias Aplicadas, Lima, Peru. Universidad Peruana de Ciencias Aplicadas Faculty of Health Sciences School of Medicine Universidad Peruana de Ciencias Aplicadas Lima Peru; 3 National Program for Youth Centers (PRONACEJ), Lima, Peru. National Program for Youth Centers (PRONACEJ) Lima Peru; 4 Department of Global Health and Social Medicine, Harvard Medical School, MA, USA. Department of Global Health and Social Medicine Harvard Medical School MA USA

**Keywords:** Tuberculosis, Youth, Adolescents, Correctional Facilities

## Abstract

This study aimed to describe the rate of tuberculosis (TB) found by using the active search strategy in teenagers and youths in three youth detention centers. TB was screened through the active search algorithm with chest X-ray, the automated reading was carried out by artificial intelligence software, the GeneXpert Ultra MTB/RIF molecular test, and clinical evaluation. A total of 640 individuals were screened, 94 (14.6%) had an abnormal chest X-ray. Of those screened, we obtained 105 GeneXpert tests of which 94 had abnormal X-rays, 9 were respiratory symptomatic and 2 were on antiretroviral treatment with TB clinical picture. We obtained 8 (8.5%) cases of TB detected with GeneXpert, 7 with abnormal radiography and 1 with normal radiography. Finally, of these 8 cases, 3 were cases of rifampicin-resistant tuberculosis (RR-TB) (42.8%). The rate of screening by active search was 1250 per 100,000 screened, 10 times higher than the rate in the general population. We recommend the inclusion of youth detention centers as target groups for systematic screening and the development of interventions to reduce the risk of TB infection.

## INTRODUCTION

Worldwide, in 2021, 10.6 million people were estimated to have tuberculosis (TB) and 1.6 million died from this cause. In the Americas, 309,000 cases of TB were estimated in 2021. A relevant fact is that TB deaths increased by 5,000 (18.5%) in 2021 compared to 2020 due to the COVID-19 pandemic ^1^.

On the other hand, people deprived of their liberty are often the last budgetary priority in any country with scarce resources. They are the extremes of the marginalized. They live in overcrowded conditions with insufficient ventilation, hygiene and sanitation. Institutional food is often scarce and not very nutritious. Illegal behaviors, such as alcohol and drug use, sexual relations (consensual or not) can occur unchecked. These conditions are conducive to chronic infectious diseases, including TB ^2^^,^^3^. This same situation occurs in Peru’s youth detention centers, which are responsible for the diagnosis and/or rehabilitation of low-income youth and adolescents in conflict with criminal law ^4^. However, these centers are inhabited by many people, have poor ventilation and overcrowded conditions that facilitate TB infection ^5^^,^^6^.

TB is the leading cause of morbidity in the prison population. It is estimated that the incidence in this population is very high, which could reach up to 7,000 cases per 100,000 persons deprived of liberty per year; on the other hand, the risk of TB infection in this population is much higher than in the general population; likewise, its impact on the general population is important since it is estimated that about 6.3% of those infected with TB occurred through contact with the population deprived of liberty ^7^.

Given the need to reduce the TB detection gap in Peru, and with the aim of actively searching for tuberculosis in vulnerable populations such as persons deprived of their liberty, the NGO Partners In Health, Peru Office (SES) and the National Program of Juvenile Centers (PRONACEJ) of the Peruvian Ministry of Justice, implemented in 2021 a strategy of active TB screening aimed primarily at youth and adolescents residing in juvenile prisons in Lima and Arequipa. This article describes the results of screening for pulmonary TB in three youth detention centers in Peru, using highly sensitive tests such as the automated interpretation system for reading chest X-rays (sensitivity ~90%) to identify persons with suspected TB, which are subsequently tested by highly specific tests such as the GeneXpert molecular test (specificity ~99%), which allows confirming the diagnosis of TB, as well as the presence of resistance to rifampicin ^8^^,^^9^^,^^10^.

## THE STUDY

This study is a secondary database analysis of an intervention based on the active search strategy for the detection of TB and rifampicin-resistant tuberculosis (RR-TB) in three youth detention centers in Peru (two in Lima and one in Arequipa) conducted by the Tuberculosis Program of Partners in Health (SES) during May to June 2021. The study population consisted of young people and adolescents deprived of their liberty. The selected centers were the Centro Juvenil de Varones de Lima (n=520), Centro Juvenil de Mujeres de Santa Margarita in Lima (n=62) and Centro Juvenil de Varones in Arequipa (n=58). All activities were carried out in coordination with the authorities of the National Program for Youth Centers (PRONACEJ) and the health area of that institution. No sample calculation was performed since the entire population was screened for TB.

We collected demographic information and applied a TB symptom self-report form. If a participant was on anti-TB treatment, they no longer went through the care flowchart. Regardless of whether they had symptoms or not, participants were voluntarily assessed by chest radiography with portable X-ray equipment (ECORAY, model Orange 1040 HF) and the radiographic images were transferred to a laptop computer and interpreted by an automated reading program (Qure.ai V2.0) using an artificial intelligence (AI) algorithm ^11^. The reading results provided normal or abnormal findings (suspicion of active TB). Subsequently, all radiographs considered abnormal were evaluated by PRONACEJ physicians. All participants with: (1) abnormal chest X-rays and/or (2) reported to be respiratory symptomatic (cough with phlegm for more than 14 days), or (3) reported to be taking antiretroviral treatment and presented clinical symptoms compatible with TB (weight loss, night sweats, fever) provided sputum samples. The samples were transported to the SES biosafety level 3 molecular diagnostic laboratory and processed using the GeneXpert® MTB/RIF Ultra molecular test ^12^.

All participants with abnormal radiographs were clinically evaluated by physicians at each youth center. The clinical evaluation in combination with chest X-ray results and GeneXpert results allowed physicians to make the diagnosis of active pulmonary TB or RR-TB with bacteriological confirmation (MTB detected with or without resistance to RIF in GeneXpert) or active pulmonary TB by clinical criteria and radiological findings. All diagnosed patients were referred to the PRONACEJ TB Program for initiation of anti-TB treatment.

All information was confidential and obtained from the SES information system (SEIS). The following indicators of the TB care cascade were reported: (1) percentage of screened population; (2) percentage of people with abnormal X-rays; (3) percentage of people with GeneXpert Ultra test; (4) percentage of people diagnosed with TB and RR-TB by GeneXpert or TB by clinical and radiological criteria. All these results were reported globally and also distributed according to each center, and we obtained the percentage of people diagnosed with TB and RR-TB from them.

All analyses were performed in the statistical program Stata/SE 16.0 (Stata Corp. College Station, TX) (13).

### Ethical Aspects

The study was approved by the SES Institutional Research Ethics Committee (approval number 0029) as a secondary source project, with unidentified data.

## FINDINGS

A total of 640 young people and adolescents deprived of their liberty were screened (520 (81.4%) from the Lima Juvenile Center for Boys; 62 (9.5%) from the Santa Margarita Juvenile Center in Lima; and 58 (9.1%) from the Arequipa Juvenile Center). The median age of the total number of participants was 20 (IQR: 2) years, with a higher percentage of people between 18 and 29 years of age (586/640, 91.6%), and males (578/640, 90.3%). Likewise, a large percentage of participants (>80%) did not report symptoms on self-report forms. Only two people reported having HIV and being currently under antiretroviral treatment. All participants from youth centers in Lima and Arequipa were male, while all participants from the Santa Margarita Youth Center in Lima were female. Both age and the presence of symptoms such as coughing or night sweats were higher in the participants of the Lima Youth Center compared to the Santa Margarita and Arequipa Youth Centers, as shown in Table 1.

**Table 1:** Sociodemographic characteristics of the participants.

Characteristics		Total N=640 (%)	Centro Juvenil de Varones de Lima n=520 (%)	Centro Juvenil de Mujeres de Santa Margarita en Lima n=62 (%)	Centro Juvenil de Varones en Arequipa n=58 (%)
Age					
	Mean (SD)	19.9 (1.8)	20.2 (1.7)	19.4 (2.1)	19.9 (1.8)
	Median (IQR)	20 (2.0)	20 (2.0)	20 (3.0)	20 (2.0)
	12 -14 years	1 (0.1)	0 (0.0)	0 (0.0)	1 (1.8)
	15 - 17 years	53 (8.3)	39 (7.5)	12 (19.4)	2 (3.4)
	18 -29 years	586 (91.6)	481 (92.5)	50 (80.6)	55 (94.8)
Sex					
	Male	578 (90.3)	520 (100.0)	0 (0.0)	58 (100.0)
	Female	62 (9.7)	0 (0.0)	62 (100.0)	0 (0.0)
TB symptoms ^a^					
	Cough	48 (7.5)	44 (8.4)	4 (6.4)	0 (0.0)
	Cough < 14 days	39 (81.2)	36 (81.8)	3 (75.0)	0 (0.0)
	Cough ≥ 14 days	9 (18.7)	8 (18.1)	1 (25.0)	0 (0.0)
	Cough with phlegm	53 (8.2)	46 (8.8)	6 (9.6)	1 (1.7)
	Cough with phlegm < 14 days	44 (83.0)	38 (82.6)	5 (83.3)	1 (100.0)
	Cough with phlegm ≥ 14 days	9 (16.9)	8 (17.3)	1 (16.6)	0 (0.0)
	Fever	32 (5.0)	30 (5.7)	2 (3.2)	0 (0.0)
	Night sweats	57 (8.9)	55 (10.5)	2 (3.2)	0 (0.0)
	Weight loss	91 (14.2)	75 (14.4)	11 (17.7)	5 (8.6)
Antiretroviral medication (TARGA) ^a^		2 (0.3)	1 (0.1)	1 (1.6)	0 (0.0)

SD: standard deviation; IQR: interquartile range; TB: tuberculosis.

a Values are not independent.

Of the 640 participants screened with a chest X-ray, 94 (14.7%) had abnormal findings, with this percentage being higher in participants from the Santa Margarita Youth Center (14/62, 22.6%) compared to the Lima Youth Center (78/520, 15.0%) and Arequipa Youth Center (2/58, 3.5%). All participants with abnormal findings on chest X-rays provided sputum samples for GeneXpert analysis, to which 11 sputum samples were added (9 samples from participants who reported symptomatology in the self-report and 2 samples from people with HIV on antiretroviral treatment). We analyzed 105 sputum samples using the GeneXpert test, of which 7 (6.7%) were positive for Mycobacterium tuberculosis (MTB); all of these samples came from participants at the Lima Youth Center. Of these 7 MTB-positive samples on GeneXpert, 3 (42.9%) showed resistance to rifampicin (Supplementary Material).

Overall, 8 persons deprived of their liberty were diagnosed with TB through the active search strategy, of which 7 had bacteriological confirmation (positive GeneXpert result) and one was diagnosed through clinical criteria and radiological findings. From these results we found that the screening rate by active search was 1250 per 100,000 screened. Of the positive TB and RR-TB cases, 75% (n=5) were asymptomatic cases.

Regarding thoracic radiographic findings and age, the highest percentage of abnormal findings was found in participants aged 15 to 17 years (18/53, 34.0%) compared to participants aged 18 to 29 years (76/586, 13.0%) and only one participant aged 12 to 14 years had abnormal radiographic findings.

## DISCUSSION

During the screening performed in youth detention centers we found high incidence of TB and RR-TB being 10 times higher than the rate in the general population, as reported by the World Health Organization ^14^, so it is necessary to include active search strategies in these places to reduce the diagnostic gap and control the transmission of the disease. We did not find studies with a similar population during the literature search. Therefore, including more active TB early detection strategies are necessary, and these have better results than classic passive TB screening strategies. In 2021, the World Health Organization (WHO) provided guidelines on systematic TB screening that include disadvantaged communities with limited access to health care such as populations deprived of their liberty, the WHO even mentions that since they have high risk of transmission it is preferable to use a highly sensitive algorithm such as the use of chest X-ray as a first step. As a second point in the algorithm, the use of rapid molecular tests should be considered, which could help to initiate TB treatment in a timely manner.

Although the entire population of youth detention centers was not screened, we found that almost half of the adolescents with TB were also found to have RR-TB (42.9%), which is almost double compared to what was found in the general community through active TB screening (~22%). Likewise, the prevalence of RR-TB in adolescents in Brazil between 2011 to 2016 was 0.5% ^15^ much lower than what was found in our study. However, no similar studies were found that include active TB search in adolescents or young people deprived of their liberty, for that reason we could not corroborate that our intervention group is more vulnerable to resistance than others.

Most of the people screened through active search (75%) did not mention having any symptoms of TB. Not feeling sick, it is likely that these individuals would not have sought medical care or been screened according to the passive TB search strategy. Global prevalence surveys report that half of sputum-positive TB cases are asymptomatic ^16^. Subclinical TB is poorly characterized, but may represent a significant proportion of TB transmission ^17^. Our results reaffirm what has been found in the literature about active search, representing a fundamental strategy that detects six times more cases than regular passive search rates ^5^^,^^8^.

A relevant finding of our study was the higher percentage of thoracic X-rays with abnormal results in females than in males, although none of these females with abnormal X-rays were positive for GeneXpert. We could not find a reason to explain these findings.

One of the limitations of the study was the sample size, which made it difficult for us to include some meaningful relations among the participants’ data.

Based on our results, we recommend conducting a cost-benefit analysis of systematic screening in the vulnerable population and encouraging institutions to implement bold policies to accelerate progress toward TB elimination. In addition, approaches that can be included within and outside the health sector should be adapted to strengthen social protection and influence the social determinants of TB, as well as to further promote TB research and innovation in this vulnerable population.

On the other hand, our findings allow us to determine that the active search algorithm we used probably detects cases earlier, and therefore improves the prognosis of the cases, in addition to reducing the chain of transmission. Therefore, it is necessary for decision makers to highlight the importance of being able to formulate new policies that contribute to the strict control of TB.

In conclusion, the active search in youth centers contributes to close the diagnostic gap that currently exists, identifying young people and adolescents without symptoms that would not be identified through passive search.

Although this active TB search strategy may be costly initially, the long-term economic benefits are significant. Early diagnosis reduces the costs associated with treatment of late-stage disease, hospitalization and community outbreaks.
