# Supplementary material for: Active search for tuberculosis in three youth detention centers in Peru
Source: Rev Peru Med Exp Salud Publica. 2024 Nov 29;41(4):417–21. doi: 10.17843/rpmesp.2024.414.13727 (PMC11797578; doi:10.17843/rpmesp.2024.414.13727)
Supplement: Supplementary material. — Available in the electronic version of the RPMESP. [file rpmesp-41-04-13727-s001.docx]

**MATERIAL SUPLEMENTARIO**

**Figura 1.** Cascada diagnóstica de TB y TB-RR en jóvenes y adolescentes privados de su libertad.

**Personas que se acercaron a las unidades móviles**

**N=640**

**Radiografias realizadas**

**N=546 (85,3%)**

**Radiografias normales +**

**personas con tos > 14 días o PVV**

**con clinica de TB**

**GeneXpert realizados**

**N=105 (16,4%)**

**GeneXpert positivos MTB**

**n=7 (6,**

**7**

**%)**

**resistentes a rifampicina**

**n=3 (42,**

**9**

**%)**

**Diagnosticados con TB**

**n=8 (8,5% con radiografía anormal)**

**Inicio de tratamiento**

**n=3 (37,5% tratamiento iniciado dentro de las 3 semanas)**

**Radiografias anormales**

**N= 94 (14,6%)**
