# Supplementary material for: Active search for tuberculosis in three youth detention centers in Peru
Source: Rev Peru Med Exp Salud Publica. 2024 Nov 29;41(4):417–21. doi: 10.17843/rpmesp.2024.414.13727 (PMC11797578; doi:10.17843/rpmesp.2024.414.13727)
Supplement: Translation

# Translation: Búsqueda activa de tuberculosis en tres centros penitenciarios juveniles de Perú

**Authors:** Judith Jimenez, Ana Karina Millones, Daniela Puma, Jesús Peinado, Blanca Martínez, Marco Tovar, Leonid Lecca

**Affiliations:** 1 Socios En Salud Sucursal Perú. Lima, Perú.; 2 Facultad de Ciencias de la Salud, Escuela de Medicina, Universidad Peruana de Ciencias Aplicadas, Lima, Perú.; 3 Programa Nacional de Centros juveniles (PRONACEJ), Lima, Perú.; 4 Department of Global Health and Social Medicine, Harvard Medical School, MA, USA.

**Keywords:** Tuberculosis, Jóvenes, Adolescentes, Centros Penitenciarios

## Abstract

El objetivo fue describir la tasa de tuberculosis (TB) hallada por la estrategia de búsqueda activa en adolescentes y jóvenes en tres centros penitenciarios juveniles. Realizamos detección de TB a través de un algoritmo de búsqueda activa con toma de radiografía de tórax, lectura automatizada por software de inteligencia artificial, prueba molecular de GeneXpert Ultra MTB/RIF, y evaluación clínica. Se tamizaron 640 personas, 94 (14,6%) tuvieron una radiografía de tórax anormal. De los tamizados, se obtuvo 105 pruebas de GeneXpert de los cuales 94 tuvieron radiografía anormal, 9 fueron sintomáticos respiratorios y 2 estuvieron en tratamiento antirretroviral con cuadro clínico de TB. Obtuvimos 8 (8,5%) casos de TB detectados con GeneXpert, 7 con radiografía anormal y 1 con radiografía normal. Por último, de estos 8 casos, 3 fueron tuberculosis resistente a rifampicina (TB-RR) (42,8%). La tasa de tamizados por búsqueda activa fue de 1250 por 100 000 tamizados, siendo 10 veces más que la tasa en población general. Recomendamos la inclusión de centros penitenciarios juveniles como grupos de interés para búsqueda sistemática y desarrollar intervenciones para disminuir riesgo de contagio de TB.

## INTRODUCCIÓN

En el mundo, en 2021, se estimó que 10,6 millones de personas padecieron de tuberculosis (TB) y 1,6 millones fallecieron por esta causa. A su vez, en las Américas, en 2021, se pudo estimar 309 000 casos de TB. Un dato relevante es que las muertes por TB aumentaron en 5000 (18,5%) en 2021 en comparación con 2020 debido a la pandemia de la COVID-19 ^1^.

Por otro lado, en cualquier país con escasos recursos, las personas privadas de su libertad suelen ser la última prioridad presupuestaria. Son los extremos de los marginados. Viven hacinadas en instalaciones con insuficiente ventilación, higiene y saneamiento. La comida institucional suele ser escasa y poco nutritiva. Los comportamientos ilegales, como el consumo de alcohol y drogas, las relaciones sexuales (consentidas o no) pueden ocurrir sin control. Estas condiciones propician enfermedades infecciosas crónicas, entre ellas la TB ^2^^,^^3^. Esta misma situación sucede en los centros penitenciarios juveniles del Perú, quienes se encargan del diagnóstico y/o rehabilitación de jóvenes y adolescentes de bajos recursos económicos y en conflicto con la Ley Penal ^4^. No obstante, estos centros son habitados por numerosas personas, tienen poca ventilación y reúnen las condiciones de hacinamiento que facilitan el contagio por TB ^5^^,^^6^.

La TB es la primera causa de morbilidad en población privada de la libertad. Se estima que la incidencia en esta población es muy alta, que podría llegar hasta los 7000 casos por cada 100 000 personas privadas de la libertad por año; de otra parte, el riesgo para contagio con TB en esta población es mucho mayor que en la población general; asimismo, su impacto en la población general es importante ya que se estima que cerca del 6,3% de los infectados por TB ocurrieron por contacto con población privada de la libertad ^7^.

Dada la necesidad de disminuir la brecha de detección de TB en el Perú, y con el objetivo de realizar la búsqueda activa de tuberculosis en esta población vulnerable como son las personas privadas de su libertad, la ONG Socios En Salud Sucursal Perú (SES) y el Programa Nacional de Centros Juveniles (PRONACEJ) del Ministerio de Justicia del Perú, implementaron el año 2021 una estrategia de búsqueda activa de TB dirigido principalmente a los jóvenes y adolescentes residentes en centros penitenciarios juveniles de Lima y Arequipa. Este artículo describe los resultados del tamizaje para TB pulmonar en tres centros penitenciarios juveniles del Perú, mediante el uso de pruebas altamente sensibles como es el sistema de interpretación automatizado para la lectura de las radiografías de tórax (sensibilidad ~90%) que permiten identificar personas con sospecha de TB, las cuales son posteriormente testeadas mediante pruebas altamente específicas como la prueba molecular de GeneXpert (especificidad ~99%), lo que permite confirmar el diagnóstico de TB, así como la presencia de resistencia a la rifampicina ^8^^,^^9^^,^^10^.

## EL ESTUDIO

El presente estudio es un análisis de bases de datos secundarios de una intervención basada en la estrategia de búsqueda activa para la detección de TB y tuberculosis resistente a rifampicina (TB-RR) en tres centros penitenciarios juveniles en Perú (dos de Lima y uno de Arequipa) realizado por el Programa de Tuberculosis de Socios En Salud (SES) durante mayo a junio del 2021. La población del estudio estuvo conformada por jóvenes y adolescentes, privados de su libertad. Los establecimientos seleccionados fueron el Centro Juvenil de Varones de Lima (n=520), Centro Juvenil de Mujeres de Santa Margarita en Lima (n=62) y el Centro Juvenil de Varones en Arequipa (n=58). Todas las actividades fueron realizadas de manera programática en coordinación con autoridades del Programa Nacional de Centros Juveniles (PRONACEJ) y el área de salud de dicha institución. No se realizó cálculo muestral ya que se dio el beneficio del descarte de TB a toda la población.

Se colectó información demográfica y aplicó una ficha de autorreporte de síntomas de TB. Si un participante se encontraba en tratamiento anti-TB, ya no pasaba por el flujograma de atención. Independientemente si presentaron síntomas o no, los participantes fueron evaluados de manera voluntaria mediante una radiografía torácica con un equipo portátil de rayos X (ECORAY, modelo Orange 1040 HF) y las imágenes radiográficas fueron transferidas a una computadora portátil e interpretada por un programa de lectura automatizada (Qure.ai V2.0) mediante un algoritmo de inteligencia artificial (IA) ^11^. Los resultados de la lectura proporcionaron hallazgos normales o anormales (sospecha de TB activa). Posteriormente todas las radiografías consideradas anormales fueron evaluadas por los médicos del PRONACEJ. Todo participante con: (1) radiografía torácica anormal y/o que (2) reportaron ser sintomáticos respiratorios (tos con flema mayor a 14 días), o (3) que haya referido estar tomando tratamiento antirretroviral y presentaban cuadro clínico compatible a TB (pérdida de peso, sudoración nocturna, fiebre) proporcionaron muestra de esputo. Las muestras fueron transportadas al laboratorio de diagnóstico molecular de nivel de bioseguridad 3 de SES y procesadas mediante la prueba molecular GeneXpert® MTB/RIF Ultra ^12^.

Todos los participantes con radiografía anormal fueron evaluados clínicamente por los médicos de cada centro juvenil. La evaluación clínica en combinación con resultados de radiografía torácica y resultados de GeneXpert permitieron que los médicos puedan realizar el diagnóstico de TB o TB-RR pulmonar activa con confirmación bacteriológica (MTB detectado con resistencia o no a RIF en GeneXpert) o TB pulmonar activa por criterio clínico y hallazgos radiológicos. Todos los pacientes diagnosticados fueron derivados al Programa de tuberculosis de PRONACEJ para su inicio de tratamiento anti-TB.

Toda la información fue confidencial y obtenida del sistema de información de SES (SEIS). Se reportaron indicadores de la cascada de cuidado TB: (1) porcentaje de población tamizada; (2) porcentaje de personas con placas radiográficas con resultado anormal; (3) porcentaje de personas en que se realizó prueba de GeneXpert Ultra; (4) porcentaje de personas diagnosticadas con TB y TB-RR mediante GeneXpert o TB mediante criterio clínico y radiológico. Todos estos resultados fueron reportados de forma global y además distribuidos según cada establecimiento y a partir de ellos se obtuvo porcentaje de personas diagnosticadas con TB y TB-RR.

Todos los análisis fueron realizados en el programa estadístico Stata/SE 16.0 (Stata Corp. College Station, TX) ^13^.

### Aspectos éticos

El estudio fue aprobado por el Comité Institucional de Ética en Investigación de SES (número de aprobación 0029) como un proyecto de uso de fuente secundaria, con datos no identificados.

## HALLAZGOS

Se tamizaron 640 jóvenes y adolescentes privados de su libertad, 520 (81,4%) del Centro Juvenil de Varones de Lima; 62 (9,5%) del Centro Juvenil Santa Margarita de Lima; y 58 (9,1%) del Centro Juvenil de Arequipa). La mediana de edad del total de participantes fue de 20 (RIC: 2) años, siendo mayor porcentaje personas entre 18 a 29 años (586/640, 91,6%), y varones (578/640, 90,3%). Asimismo, un gran porcentaje de participantes (>80%) no reportaron síntomas en fichas de autorreporte. Únicamente, dos personas reportaron presentar VIH y estar actualmente bajo tratamiento antirretroviral. Todos los participantes de centros juveniles de Lima y Arequipa fueron varones, mientras que todos los participantes del Centro Juvenil de Santa Margarita de Lima fueron mujeres. Tanto, la edad como la presencia de síntomas como tos o sudoración nocturna fueron más altas en los participantes del Centro Juvenil de Lima en comparación a los centros juveniles de Santa Margarita y Arequipa, como se observa en la Tabla 1.

**Tabla 1:** Características sociodemográficas de los tamizados.

Características		Total N=640 (%)	Centro Juvenil de Varones de Lima n=520 (%)	Centro Juvenil de Mujeres de Santa Margarita en Lima n=62 (%)	Centro Juvenil de Varones en Arequipa n=58 (%)
Edad					
	Media (DE)	19,9 (1,8)	20,2 (1,7)	19,4 (2,1)	19,9 (1,8)
	Mediana (RIC)	20 (2,0)	20 (2,0)	20 (3,0)	20 (2,0)
	12 -14 años	1 (0,1)	0 (0,0)	0 (0,0)	1 (1,8)
	15 - 17 años	53 (8,3)	39 (7,5)	12 (19,4)	2 (3,4)
	18 -29 años	586 (91,6)	481 (92,5)	50 (80,6)	55 (94,8)
Sexo					
	Varón	578 (90,3)	520 (100,0)	0 (0,0)	58 (100,0)
	Mujer	62 (9,7)	0 (0,0)	62 (100,0)	0 (0,0)
Síntomas de TB ^a^					
	Tos	48 (7,5)	44 (8,4)	4 (6,4)	0 (0,0)
	Tos < 14 días	39 (81,2)	36 (81,8)	3 (75,0)	0 (0,0)
	Tos ≥ 14 días	9 (18,7)	8 (18,1)	1 (25,0)	0 (0,0)
	Tos con flema	53 (8,2)	46 (8,8)	6 (9,6)	1 (1,7)
	Tos con flema < 14 días	44 (83,0)	38 (82,6)	5 (83,3)	1 (100,0)
	Tos con flema ≥ 14 días	9 (16,9)	8 (17,3)	1 (16,6)	0 (0,0)
	Fiebre	32 (5,0)	30 (5,7)	2 (3,2)	0 (0,0)
	Sudoración nocturna	57 (8,9)	55 (10,5)	2 (3,2)	0 (0,0)
	Baja de peso	91 (14,2)	75 (14,4)	11 (17,7)	5 (8,6)
Toma de medicamentos antirretrovirales (TARGA) ^a^		2 (0,3)	1 (0,1)	1 (1,6)	0 (0,0)

DE: desviación estándar, RIC: rango intercuartílico, TB: tuberculosis.

a Los valores no son independientes.

De los 640 participantes tamizados usando una radiografía de tórax, 94 (14,7%) tuvieron hallazgos anormales, siendo este porcentaje mayor en participantes del Centro Juvenil de Santa Margarita (14/62, 22,6%) en comparación al Centro Juvenil de Lima (78/520, 15,0%) y Centro Juvenil de Arequipa (2/58, 3,5%). Todos los participantes con hallazgos anormales en radiografías torácicas proporcionaron muestras de esputo para análisis de GeneXpert, a los cuales se adicionaron 11 muestras de esputo (9 muestras de participantes que reportaron sintomatología en el autorreporte y 2 muestras de personas con VIH en tratamiento antirretroviral). 105 muestras de esputo fueron analizadas mediante la prueba de GeneXpert, de las cuales siete (6,7%) tuvieron resultado positivo para *Mycobacterium tuberculosis* (MTB)*;* todas estas muestras provenían de los participantes del Centro Juvenil de Lima. De estas 7 muestras positivas a MTB en GeneXpert*,* 3 (42,9%) demostraron resistencia a rifampicina (Material suplementario).

 En total, 8 personas privadas de su libertad fueron diagnosticadas con TB mediante la estrategia de búsqueda activa, de los cuales 7 tuvieron confirmación bacteriológica (resultado GeneXpert positivo) y uno fue mediante criterio clínico y hallazgos radiológicos. A partir de estos resultados encontramos una tasa de tamizados por búsqueda activa fue de 1250 por 100 000 tamizados. De los casos positivos de TB y TB-RR un 75% (n=5) de ellos fueron casos asintomáticos.

Con respecto a los resultados de radiografía torácica y la edad, el mayor porcentaje de hallazgos anormales se observó en participantes entre 15 a 17 años (18/53, 34,0%) en comparación a participantes entre 18 a 29 años (76/586, 13,0%) y solo un participante entre 12 a 14 años tuvo hallazgos radiográficos anormales.

## DISCUSIÓN

Durante el tamizaje realizado en los centros penitenciarios juveniles encontramos una alta incidencia de TB y TB-RR siendo 10 veces más alta que la tasa en la población general, según lo informado por la Organización Mundial de la Salud ^14^, por lo que es necesario incluir estrategias de búsqueda activa en estos lugares para reducir la brecha diagnóstica y controlar la transmisión de la enfermedad. Durante la búsqueda bibliográfica no se encontraron estudios con una población similar. Por lo que, incluir estrategias más activas de detección temprana de la TB son necesarias y tienen mejores resultados que las estrategias clásicas de búsqueda pasiva de TB. En el 2021, la Organización Mundial de la Salud (OMS) brindó directrices sobre el tamizaje sistemático de la TB que incluyen comunidades desfavorecidas con acceso limitado a la atención de salud como son las poblaciones privadas de su libertad, incluso la OMS menciona que por tener un riesgo alto de transmisión es preferible el empleo de un algoritmo sumamente sensible como el uso de radiografía de tórax como primer paso. Como segundo punto en el algoritmo se debe considerar el uso de las pruebas moleculares rápidas, las que podrían ayudar a iniciar tratamiento para la TB oportunamente.

 A pesar de que no se tamizó a toda la población de los centros penitenciarios juveniles, se observó que casi la mitad de los adolescentes identificados con TB tuvieron además resultado con TB-RR (42,9%), el cual es casi el doble comparado con lo hallado en comunidad en general a través de búsqueda activa de TB (~22%). Asimismo, en Brasil la prevalencia de TB-RR en adolescentes entre el 2011 al 2016 fue 0,5% ^15^ mucho menor a lo encontrado en nuestro estudio. Sin embargo, no se encontraron estudios similares que incluya la búsqueda activa de TB en adolescentes o jóvenes privados de su libertad, por tal motivo no podría corroborar que nuestro grupo de intervención es más vulnerable a resistencia que otros.

 Las personas tamizadas a través de búsqueda activa en su mayoría (75%) no mencionaron tener algún síntoma de TB. Al no sentirse enfermos es probable que estas personas no hubieran buscado atención médica ni hubieran sido tamizados de acuerdo con la estrategia de búsqueda pasiva de TB. Las encuestas de prevalencia mundial informan que la mitad de los casos de TB con esputo positivo son asintomáticos ^16^. La TB subclínica está mal caracterizada, pero puede representar una proporción significativa de transmisión de TB ^17^. Nuestros resultados reafirman lo encontrado en la literatura acerca de la búsqueda activa, representando una estrategia fundamental que detecta seis veces más casos que las tasas regulares de búsqueda pasiva ^5^^,^^8^.

 Un hallazgo relevante de nuestro estudio fue el mayor porcentaje de radiografías torácicas con resultados anormales en mujeres que en varones, aunque ninguna de estas mujeres con radiografías anormales resultó positiva a GeneXpert. No hemos podido encontrar una razón que explique estos hallazgos.

Una de las limitaciones del estudio fue el tamaño de muestra, lo que dificultó que incluyamos algunas relaciones significativas entre los datos de los participantes.

En base a los resultados obtenidos, recomendamos realizar un análisis del costo beneficio de un tamizaje sistemático en la población vulnerable y fomentar en las instituciones la instauración de políticas audaces para acelerar el progreso hacia la eliminación de la TB. Además, adaptar enfoques que pueden incluirse dentro y fuera del sector de la salud para reforzar la protección social e incidir en los determinantes sociales de la tuberculosis, así como para seguir fomentando la investigación y la innovación en tuberculosis en esta población vulnerable.

Por otro lado, los hallazgos obtenidos nos permiten determinar que el algoritmo de búsqueda activa que usamos probablemente detecta casos de forma más temprana, y por ende mejora el pronóstico de los casos, además que disminuye la cadena de transmisión. Por ello, es necesario que los tomadores de decisiones resalten la importancia de poder formular nuevas políticas que contribuyan al control estricto de la TB.

En conclusión la búsqueda activa en centros juveniles contribuyen a cerrar la brecha diagnóstica que actualmente existe, identificando jóvenes y adolescentes sin síntomas los cuales no serían identificados a través de la búsqueda pasiva.

Aunque esta estrategia de búsqueda activa de tuberculosis pueda resultar ser costosa en un inicio, los beneficios económicos a largo plazo son significativos. El diagnóstico temprano reduce los costos asociados con el tratamiento de la enfermedad en sus etapas avanzadas, la hospitalización y los brotes comunitarios.
